# Remodeling of algal photosystem I through phosphorylation

**DOI:** 10.1042/BSR20220369

**Published:** 2023-01-24

**Authors:** Muhammad Younas, Martin Scholz, Giulia Maria Marchetti, Michael Hippler

**Affiliations:** 1 Institute of Plant Biology and Biotechnology, University of Münster, 48143 Münster, Germany; 2 Institute of Plant Science and Resources, Okayama University, Kurashiki, Japan

**Keywords:** green algae, Light harvesting proteins, phosphorylation/dephosphorylation, photosystems

## Abstract

Photosystem I (PSI) with its associated light-harvesting system is the most important generator of reducing power in photosynthesis. The PSI core complex is highly conserved, whereas peripheral subunits as well as light-harvesting proteins (LHCI) reveal a dynamic plasticity. Moreover, in green alga, PSI–LHCI complexes are found as monomers, dimers, and state transition complexes, where two LHCII trimers are associated. Herein, we show light-dependent phosphorylation of PSI subunits PsaG and PsaH as well as Lhca6. Potential consequences of the dynamic phosphorylation of PsaG and PsaH are structurally analyzed and discussed in regard to the formation of the monomeric, dimeric, and LHCII-associated PSI–LHCI complexes.

## Introduction

**Figure 1:**
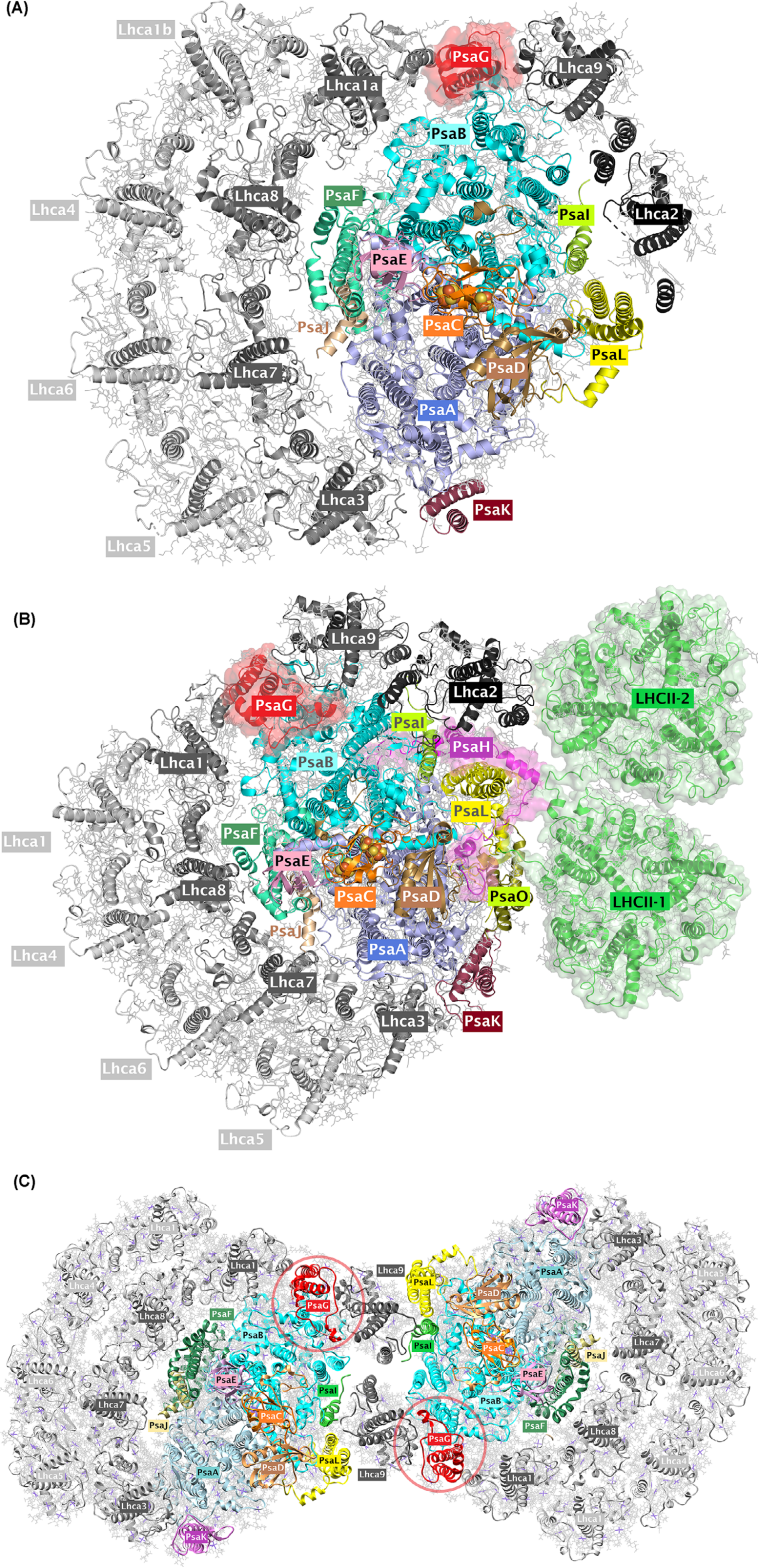
An overview of PsaG and/or PsaH subunits in monomeric, dimeric, and state transition complex of PSI (**A**) Cartoon representation of the *Chlamydomonas* PSI with ten Lhca (Protein Data Bank (PDB) ID: 6JO5). Subunits are color-coded. PsaG subunit is highlighted in surface. (**B**) Cartoon representation of the *Chlamydomonas* PSI coupled with additional LHCII trimers during state transition (PDB ID: 7DZ7). Subunits are color-coded. PsaG and PsaH subunits are highlighted in surface. (**C**) Cartoon representation of the *Chlamydomonas* PSI dimer (PDB ID: 7ZQD). Subunits are color-coded. PsaG subunits are marked in red circles.

The core of photosystem I (PSI) is highly conserved from cyanobacteria to vascular plants [1–4]. The light-driven electron transfer at PSI is certainly the most important generator of reducing power at cellular level. PSI consists of more than ten subunits and many different cofactors such as chlorophyll (Chl) *a*, β-carotene, phylloquinone, and three (4Fe-4S) clusters. Light-induced primary charge separation occurs between the primary electron donor (P700) and acceptor (A_0_). The reduced A_0_ donates an electron to iron–sulfur cluster F_X_ via phylloquinone (A_1_). These redox components as well as most antenna pigments are located on a heterodimer of the two homologous subunits encoded by the chloroplast *psaA* and *psaB* genes. Reduced F_X_ forwards an electron to iron–sulfur cluster F_A_ and subsequently to the other iron–sulfur cluster F_B_ that reduces ferredoxin (FDX) as the terminal electron acceptor of PSI. FDX in turn reduces the FDX-NADP-reductase (FNR), leading to the formation of NADPH. The core of PSI further harbors approximately 100 Chls [2], which serve as antenna system to collect light energy. This core antenna is extended by additional Chl-binding proteins that form the light-harvesting complex (LHCI). During PSI evolution, heterodimerization established specific peripheral-binding sites that are unique for each monomer, though the center is tightly conserved [5]. For example, in cyanobacterial PSI, PsaK binds exclusively to PsaA [6]. On the other hand, in eukaryotic green lineage PSI, PsaG, a homolog of PsaK, binds exclusively to PsaB. Also, other peripheral subunits bind to their neighbors in an asymmetric fashion. This type of plasticity is further observed for binding of LHCI to PSI. High-resolution structures from vascular plants revealed that PSI contains four LHCs [7–10], while PSI of green algae may contain up to ten LHCs, two of which at the PsaL pole and up to eight arranged in two crescents at the PsaF pole (pointing toward the lumen) (Figure 1A) [11–15]. Notably, also in *Arabidopsis thaliana*, an additional Lhca1-a4 dimer was found to be bound on the PsaL side [16], suggesting that this mode of organization is present in vascular plants. In this low-resolution complex, also a LHCII trimer with contact to an additional Lhca1-a4 dimer at the PsaL side was also present.

Binding of LHCII to PSI is a common incidence in most organisms of the green lineage and driven by a process called state transitions [17]. State transitions are important to balance the excitation energy between PSI and PSII [18,19]. Under light conditions where PSII is preferentially excited, both PSII core and LHCII proteins become phosphorylated [20]. As a consequence, phosphorylated LHCII proteins detach from PSII and partly connect to PSI (state II). Under conditions where PSI excitation is predominant, this process is reversed. LHCII proteins are dephosphorylated and associate to PSII (state I) [21]. The kinase responsible for LHCII phosphorylation is STT7 in *Chlamydomonas reinhardtii* [22] or STN7 in *A. thaliana* [23]. High-resolution cryogenic electron microscopy (cryo-EM) structures of PSI–LHCI–LHCII complexes representing state II transition were revealed via cryo-EM from maize [24] and *C. reinhardtii* [25] (Figure 1B). In both structures, phosphorylated LHCII could be visualized being in contact with peripheral PSI subunits, particularly PsaH and PsaL, indicating that the recognition pattern between the phosphorylated LHCII-1 trimer and the PSI core is conserved in the plant lineage [25]. Notably, the PSI–LHCI–LHCII complex from *C. reinhardtii* contains two LHCII trimers (termed as LHCII-1 and LHCII-2) [25]. The LHCII-2 trimer associates with PSI via LhcbM5. LhcbM5 is phosphorylated and its phosphorylation depends on STT7 [26]. LhcbM5 phosphorylated at T33 binds K60 of PsaH [25]. Moreover, residues R35 and N43 interact with E58 and D64 from PsaH [25]. These three PsaH residues are not conserved in maize PsaH, thus indicating that the PSI-binding site for the LHCII-2 trimer is algal specific. Besides PsaH, LhcbM5 interacts extensively with Lhca2 through its AC and BC loop regions.

In a recent cryo-EM study, Naschberger et al. [27] identified a PSI dimer from *C. reinhardtii* where two copies of Lhca9 tethered two monomeric PSI in a head-to-head fashion, forming a large oligomeric protein complex (Figure 1C). Notably, in this dimeric PSI structure, Lhca2 and PsaH are absent [27]. The dimer is stabilized by four types of interactions that cover the entire membrane span: (i) a hydrogen bond of the backbone carbonyl of G148 with S137 of PsaL in the stroma; (ii) a hydrogen bond between the two Q109 of the Lhca9 copies; (iii) hydrophobic contacts via coordinated cofactors in the membrane that include a newly modeled, β-carotene 623 and five Chls (604, 610, 611, 612, and 810); (iii) lipid-mediated hydrophobic interactions via monogalactosyl diglyceride LMG852 and LMU624 [27]. Interestingly, an atomic force microscopy analysis of the membrane architecture in dark- and light-adapted membranes of *A. thaliana* revealed ordered rows of closely packed PSI dimers, which are more abundant in the dark state [28]. Likewise, closely associated PSI–LHCI complexes were identified in solubilized membranes of *A. thaliana* by negative stain electron microscopy [29]. Cryo-EM analyses of PSI particles from a temperature-sensitive PSII mutant *Chlamydomonas* strain also showed the presence of dimers [30]. A reversible PSI dimer formation may have a physiological role in thylakoid membrane structure maintenance in chloroplasts. Importantly, the formation of PSI–LHCI–LHCII and PSI dimers are mutually exclusive, as dimer formation would clash with structural features of the state transition complex [27].

How could this dynamic remodeling that transform the monomeric PSI–LHCI to either dimeric PSI or state transition complex (PSI–LHCI–LHCII) be envisioned? Possible key players in the remodeling regulation are likely PsaH and Lhca2. PsaH is an 11-kDa transmembrane protein that is imported into chloroplasts and peripherally associates with the PSI reaction center on the opposite side to the LHCI belt [2]. During PSI biogenesis, PsaH is assembled in a separate protein module that occurs before Lhca2 assembly [31]. PsaH knockout mutants in *A. thaliana* can grow photoautotrophically [32]. Moreover, structures of functional algal PSI particles that lack PsaH have also been determined [11,33]. A recent proteome analysis of the pyrenoid tubules from *C. reinhardtii* revealed an enrichment of PsaH, whereas no other PSI subunits were detected [34]. All these data suggest the presence of PsaH-lacking PSI complexes *in vivo*. Thus, the absence of PsaH/Lhca2 could induce the PSI dimer formation where PSI monomers associate with each other to form larger complexes. This feature is likely to be conserved in plants, as PSI fractions bigger than monomeric PSI have been reported [35]. The presence of LHCI-dimer at the PsaL pole in *A. thaliana* [16] may indicate that a similar mechanism drives PSI dimer formation in vascular plants. Since the basic structure of the PSI core is rigid, the regulation by PsaH/Lhca2 provides a degree of plasticity that would allow stress acclimation due to PSI remodeling and tolerate changes in the range of light intensities that might involve membrane reorganization [28,36]. Thus, the PsaH/Lhca2 module could regulate PSI dimer formation and co-ordinate the macromolecular organization of PSI in chloroplasts. This regulatory role could be mediated through post-translational modifications such as phosphorylation in *C. reinhardtii* [26] or acetylation in *A. thaliana* [37]. In *C. reinhardtii*, a consequence of PsaH regulation would be the differential binding of Lhca2. There is evidence that PSI oligomerization through PSI dimer formation could regulate electron partitioning at the PSI acceptor side [38]. It is proposed that, under anoxia, PSI remodeling and dimer formation channel electron efficiently to hydrogenase, thereby modulating electron partitioning in favor of H_2_ production, which in turn increases the sink capacity for photosynthetic electrons. This is based on the data obtained with *lhca2/pgr5* double mutant [38], which produced significantly more H_2_ as compared with *lhca2* and *pgr5* single mutants as well as *lhca9/pgr5* double mutant and *pgr5/lhca2/lhca9* triple mutant. In the *lhca2/pgr5* double mutant, Lhca9 is present and could stimulate dimer formation in the absence of Lhca2. Thus, engineering of PSI oligomerization could alter electron partitioning at the PSI acceptor side in favor of H_2_ production. Besides PsaH, also PsaG is dynamically phosphorylated [26]. In the *C. reinhardtii* PSI structure, PsaG is interposed between Lhca1 and Lhca9 [11]. When PsaG is depleted or deleted via genetic approaches, vascular plants are still photo-autotrophic, despite an about 40–50% diminishment of PSI [39,40]. Notably, in the dimeric as well as in the state transition, but not in the monomeric PSI structure, two loops in the stromal side of PsaG and Lhca9 could be interpreted, likely due to the stabilization by the adjacent monomer in the PSI dimer [27]. This indicates that both PsaG and Lhca9 undergo a structural rearrangement upon dimer and state transitions complex formation, which is absent in the monomer.

In the current manuscript, we revisited PsaH and PsaG phosphorylation and reveal that in three independent WT strains, phosphorylation is significantly up-regulated for both proteins. The impact of PsaH and PsaG protein phosphorylation on structure and function of PSI is discussed.

## Materials and methods

### Strains and culture conditions

*C. reinhardtii* strains CC-124, CC-125, and CC-4375 IFT46::YFP [41] were grown in acetate-free Tris-phosphate (TP) liquid medium [42] on a shaker at a light intensity of 60 µmol photons m^−2^ s^−1^ under a 16 h light/8 h dark cycle. One day before the experiment, cells were diluted to 3 µg Chl/ml. Normal light (NL) samples were taken 3 h after the onset of light (NL) and centrifuged for 5 min at 2500× ***g*** to pellet the cells. The remaining cultures were diluted to 3 µg Chl/ml and transferred to continuous high light (HL; 500 µmol photons m^−2^ s^−1^). HL samples were taken after 24 h as described above. The experiment included four replicates per strain and light condition.

### Protein isolation and tryptic digestion

To disrupt the cells and extract proteins, lysis buffer (100 mM Tris-HCl (pH 8.5)/1 mM benzamidine/1 mM PMSF/10 mM sodium fluoride/1 mM sodium orthovanadate/10 mM sodium pyrophosphate/10 mM β-glycerophosphate/2% (w/v) SDS) was added to the cell pellets and samples were incubated for 15 min at 65°C in a Thermomixer at 1000 rpm, followed by centrifugation at 20000× ***g*** for 10 min to pellet cell debris. Protein concentration in the supernatant was determined by BCA assay (Pierce BCA Protein Assay Kit, Thermo Fisher Scientific). A lysate volume corresponding to 100 µg of protein was tryptically digested according to a modified FASP (Filter-aided sample preparation) protocol [43]. Briefly, cell lysates were transferred to centrifugal ultrafiltration devices (Amicon Ultra, 0.5 ml, 30 kDa cutoff, Merck Millipore) and concentrated to 20 µl by centrifugation for 20 min at 14000× ***g*** at room temperature. SDS removal and alkylation of cysteines was achieved by addition of 200 µl of 100 mM Tris-HCl/8 M urea/10 mM TCEP/40 mM chloroacetamide, followed by centrifugation as described above. The detergent removal and alkylation step were repeated three times. Subsequently, samples were washed four times with 200 µl of 100 mM ammonium hydrogencarbonate. Then, trypsin (sequencing grade, Promega) was added to the filters at a protein-to-enzyme ratio of 50:1. Digestion was carried out overnight at 37°C. Peptides were harvested by centrifugation and trifluoroacetic acid (TFA) was added to a final concentration of 0.5%.

Peptides were desalted using C18 membranes (7 mm diameter), cut from Empore C18 extraction membranes (Sigma) using a stencil, and inserted into MobiCol spin columns, sandwiched between two frits (10 µm pore size, MobiCol). The columns were conditioned and equilibrated using 80% (v/v) acetonitrile/0.1% TFA and 0.1% TFA, respectively. Then, the samples were loaded on the columns followed by centrifugation for 2–5 min at 1000× ***g***. Membranes were washed twice with 200 µl of 0.1% TFA before peptides were eluted in 100 µl of 60% (v/v) acetonitrile/0.1% TFA. Eluates were briefly mixed and each sample was split into two aliquots: a sample volume corresponding to 10 µg of peptides was used directly for characterization of the proteome (‘nonenriched’), whereas the remaining 90 µg was subjected to phosphopeptide enrichment prior to LC-MS/MS analysis (‘enriched’).

### Phosphopeptide enrichment

Phosphopeptides were enriched by metal oxide affinity chromatography (MOAC) using TiO_2_ (Titansphere, 5 µm particle size, GL Sciences). TiO_2_ (1 mg per sample) was activated once with acetonitrile and equilibrated three times with loading buffer (LB, 80% (v/v) acetonitrile/5% (v/v) TFA/1 M glycolic acid). Then, LB was added to obtain 10% (w/v) TiO_2_ slurry. Peptide samples were resuspended in 50 µl of LB, then added to 10 µl of TiO_2_ slurry and the mixture was incubated for 1 h at room temperature at 1200 rpm in a Thermomixer (Eppendorf). Afterward, the slurry was transferred to in-house made C18 STAGE tips (Empore C18, Sigma [44]). The following steps were carried out in a centrifuge (1000× ***g*** at room temperature). Buffer volume was 80 µl throughout. The buffer was removed and the TiO_2_ beads were washed twice each with LB and 1% (v/v) acetonitrile/0.1% (v/v) TFA (W1). Nonphosphorylated peptides that bound to the C18 plug were removed by two washes with 80% (v/v) acetonitrile/0.1% (v/v) TFA (W2). Weakly and moderately strongly bound phosphopeptides were eluted from the TiO_2_ and onto the C18 material by six successive washes with 0.4 M NaH_2_PO_4_/1% (v/v) acetonitrile/0.1% TFA. Phosphopeptides were washed once with W1 and subsequently eluted with 60% (v/v) acetonitrile/0.1% (v/v) TFA (fraction 1). Strongly bound phosphopeptides were eluted with 5% ammonia (fraction 2). Phosphopeptides that had detached from TiO_2_ by the ammonia treatment and bound to the C18 plug despite the high pH were washed once with W1, followed by elution with 60% (v/v) acetonitrile/0.1% (v/v) TFA (fraction 3). All fractions were dried by vacuum centrifugation and stored at –80°C until further use.

### Mass spectrometry and data analysis

Dried peptide samples were reconstituted in 5 µl of 2% (v/v) acetonitrile/0.05% (v/v) TFA in LC/MS grade water. Samples were analyzed on an LC-MS/MS system consisting of an Ultimate 3000 NanoLC (Thermo Fisher Scientific) coupled via a Nanospray Flex ion source (Thermo Fisher Scientific) to a Q Exactive Plus mass spectrometer (Thermo Fisher Scientific). Peptides were concentrated on a trap column (Acclaim Pepmap C18, 5 × 0.3 mm, 3 µm particle size, Thermo Scientific) for 3 min using 4% (v/v) acetonitrile/0.05% (v/v) in LC/MS grade water at a flow rate of 10 µl/min. The trap column was operated in back-flush mode, allowing the transfer of peptides on a reversed-phase column (Acclaim PepMap, 500 × 0.075 mm, 2 µm particle size, Thermo Fisher Scientific) for chromatographic separation. The buffers used were 0.1% (v/v) formic acid in ultrapure water (A) and 0.1% (v/v) formic acid/80% (v/v) acetonitrile in ultrapure water (B). The gradient was programmed as follows: 5% B to 36% over 160 min, 36% to 99% B over 10 min, and 99% B for 20 min. Flow rate was 300 nl/min. The mass spectrometer was operated in data-dependent acquisition mode (‘Top12’), alternating between one MS1 full scan and up to 12 MS2 scans. Full scans were acquired with the following settings: AGC target 3e6, MS1 resolution 70000, maximum injection time 50 ms, scan range: 300–1400 m/z. Settings for MS were: AGC target 5e4, resolution 17500, MS2 maximum injection time 80 ms (200 ms for enriched samples), intensity threshold 1e4, normalized collision energy 27 (HCD)).

MS raw data obtained from nonenriched and enriched samples were searched separately with MaxQuant (v. 2.0.3.0, [45]) against a concatenated database consisting of nuclear- (v. 5.6, Phytozome 13), chloroplast- (GenBank BK000554.2), and mitochondria-encoded (GenBank NC_001638.1) proteins. False discovery rates were 1% on peptide and protein level. Default settings for identification of tryptic peptides and label-free quantification (LFQ) were applied with the following exceptions: ‘Fast LFQ’ was deactivated. Phosphorylation of serine (S), threonine (T), and tyrosine (Y) were set as variable modifications in addition to oxidation of methionine. Moreover, ‘match between runs’ was enabled. Two parameter groups with identical settings were used to allow for separate normalization of LFQ data of NL and HL samples.

Protein LFQ intensity and phosphorylation site intensity data were preprocessed in Perseus (v. 1.6.15.0, [46]): Intensities were log2-transformed, followed by removal of reverse hits, contaminants, and proteins identified exclusively by modified peptides (‘only identified by site’). Median subtraction was performed for intensity normalization. Then, the lowest intensity value was subtracted from all other values of the same sample to obtain positive values only. Proteins and phosphorylation sites with less than 75% valid values in at least one condition (NL or HL) were discarded. Missing values were imputed using the impSeqRob (protein intensities) and MinProb (phosphorylation site intensities) algorithms available in the online tool NAGuideR [47–49]. Differential expression analysis was carried out using PolyStest [50] with default settings. The intensity data of CC-124, CC-125, and CC-4375 IFT46::YFP were treated as equally weighted, independent replicates, resulting in a total of 12 replicates per light condition. The mass spectrometry proteomics data have been deposited to the ProteomeXchange Consortium (http://proteomecentral.proteomexchange.org) via the PRIDE partner repository [51] with the dataset identifier PXD036166 and DOI 10.6019/PXD036166.

### Structural models

Structural models were generated using PyMOL (Schrödinger, LLC; Windows, v2.0), UCSF Chimera (Windows, v1.14; [52]), ChimeraX (Windows, v1.4; [53,54]), and BIOVIA Discovery Studio Visualizer (BIOVIA, Dassault Systèmes, Discovery Studio Visualizer, v21.1.0.20298, San Diego: Dassault Systèmes, 2021). The phosphate groups were added to the residues of interest using PyTMs [55]—a plugin designed for PyMOL for the introduction of common post-translational modifications to protein models. The interactions between amino acids were determined using PyMOL and Discovery Studio Visualizer as well as visual inspection. Multiple sequence alignment was carried out by means of Clustal Omega and the final results were displayed and analyzed using Jalview (Windows, v2.11.2.3). The reference structures employed in the manuscript were retrieved from PDB, including the structures of *Chlamydomonas* PSI dimer (PDB ID: 7ZQD), PSI-10Lhca (PDB IDs: 6JO5 and 6IJO), and PSI–LHCI–LHCII (PDB ID: 7DZ7).

## Results and discussion

**Figure 2:**
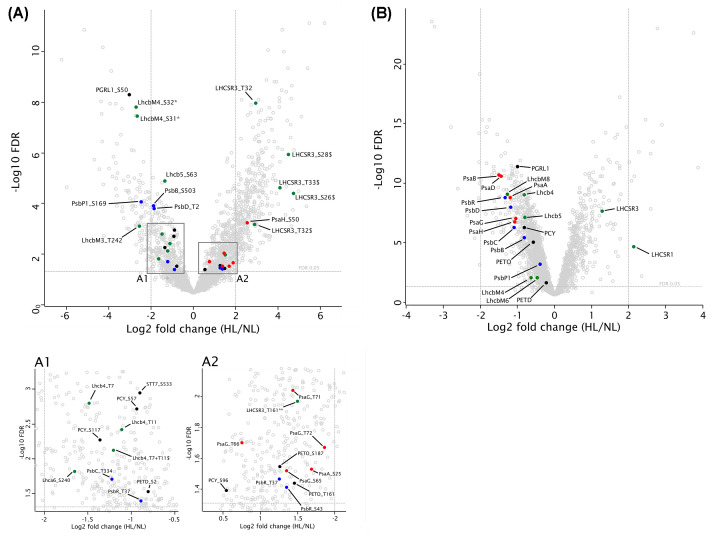
Influence of different light conditions on the abundance and phosphorylation of photosynthetic thylakoid membrane proteins Volcano plots of FDR (-log10) against log2 fold changes (HL/NL), illustrating differences in phosphorylation ( **A**) and protein (**B**) levels. Differential expression analysis was performed using Limma. Color code: PSII (blue), PSI (red), LHC (green), others (black). $, doubly phosphorylated peptides (multiplicity = 2); *, nonproteotypic peptide (LhcbM4/6/8); **, nonproteotypic peptide (LHCSR1/3). Detailed data is available in Supplementary Table S1. For each light condition, abundance data of four replicates of each of the strains CC-124, CC-125, and CC-4375 IFT46::YFP were treated as equally weighted, independent replicates (*n*=12).

We were interested in light-dependent regulatory processes that may affect protein–protein interactions within the photosynthetic protein complexes and therefore compared the phosphoproteomes of *C. reinhardtii* grown under NL and HL conditions. Here, our main focus was on PSI phosphorylation. To analyze photosynthetic protein phosphorylation, the algae were cultivated photo-autotrophically in TP medium under a diurnal light regime (16 h light/8 h dark) in NL (60 µmol photons m^−2^ s^−1^) for 1 week and then shifted to continuous HL (500 µmol photons m^−2^ s^−1^). Samples were taken right before and 24 h after the transfer. After tryptic digestion, peptide samples were split: one aliquot was analyzed directly by LC-MS/MS to obtain information about changes in protein abundances, while another aliquot underwent phosphopeptide enrichment for subsequent phosphoproteomic analysis. To identify differences in protein abundances and phosphorylation that were independent of a genetic background, three *C. reinhardtii* strains were used in the experiment (CC-124, CC-125, and CC-4375) and quantitative proteomics data of all strains were combined. In total, 12001 phosphorylation sites from 3337 proteins were identified. After filtering, 2403 quantified phosphorylation sites remained. Differential expression analysis using Limma [56] (implemented in PolySTest [57]) revealed 861 up-regulated and 442 down-regulated phosphorylation sites in HL-treated samples (false discovery rate [FDR]<0.05). Differentially phosphorylated sites include 54 sites from 23 proteins involved in the photosynthetic electron transfer machinery (Figure 2, Supplementary Table S1). These sites were already previously identified [26,57] and are therefore robust and reliable.

Phosphorylation of PSI subunits PsaG (S65, T66, T71, and T72) and PsaH (S50) increased from 1.7 to 5.7-fold in response to HL (Figure 2A, Supplementary Table S1). In contrast, protein levels of these proteins decreased by about 50%, implying a strong increase in the degree of phosphorylation (Figure 2B, Supplementary Table S1). Likewise, PsaA abundance decreased to 43% of NL levels after HL treatment, whereas, at the same time, phosphorylation of its residue S25 increased 3.2-fold. Total plastocyanin (PCY1) was diminished almost to the same extent as PsaG and PsaH. The same applies to the phosphorylation of its residue S57. Interestingly, the abundances of phosphorylated S96 and S117 of plastocyanin showed a different behavior. The former increased 1.5-fold in HL, whereas the latter decreased to less than 40% compared with NL levels. In HL, the cytochrome b_6_f subunit PETO changed in abundance similarly to PSI subunits. The amounts of PETO T161 and S187 residues, which also correspond to a STT7-dependent phosphorylation [26], however, were 2.4- and 2.7-fold higher under these conditions. Also, PGRL1 expression almost halved in HL-treated cells, yet phosphorylation of S50 decreased to around 12% of the NL level. Hence, the absolute amount of PGRL1 phosphorylated at S50 was reduced by 75% in HL. As expected [58], light stress led to an enhanced expression of LHCSR3, which becomes evident from the almost 2.4-fold higher protein abundance in HL. However, at the same time, phosphorylation levels of the N-terminal threonine residues of LHCSR3 (T32, T33) increased 7.7-fold. The changes in the amounts of N-terminal phosphopeptides bearing multiple phosphorylations were even more pronounced. For example, phosphorylation of S26 with concomitant phosphorylation of either S28, T32, or T33 was more than 26 times more abundant in HL than in NL. As shown before, these residues are the target of STT7 [26]. Accordingly, not only the absolute amount of LHCSR3 but also its relative phosphorylation levels were enhanced in HL. The degree of C-terminal phosphorylation (T161) of LHCSR3, which is phosphorylated in an STT7-independent manner [26], remained almost unchanged. It must be noted that the corresponding peptide is nonproteotypic and is shared with LHCSR1. PSBR expression was reduced by about 60% in HL, while phosphorylation of its residues T37 and S43 increased about 2.5-fold. Other phosphorylated PSII proteins were less abundant in HL as well, with decrease from 24% (PsbP1) to about 55% (PsbB, PsbC, PsbD). The abundances of most of their phosphorylations were either similarly reduced or changes were not significant. However, phosphorylation of T2 of PsbD (–73%), S136 of PsbO (+77%), and S169 of PsbP1 (–82%) clearly deviated from this trend. The PSII antenna proteins LhcbM3, LhcbM4, Lhcb4, and Lhcb5 were also diminished in NL by almost 50%. The effect on most of their phosphorylation sites was somewhat more pronounced, with S242 of LhcbM3 showing the greatest decrease to more than 80%.

### Potential functional insights of PsaG and PsaH phosphorylation

**Figure 3:**
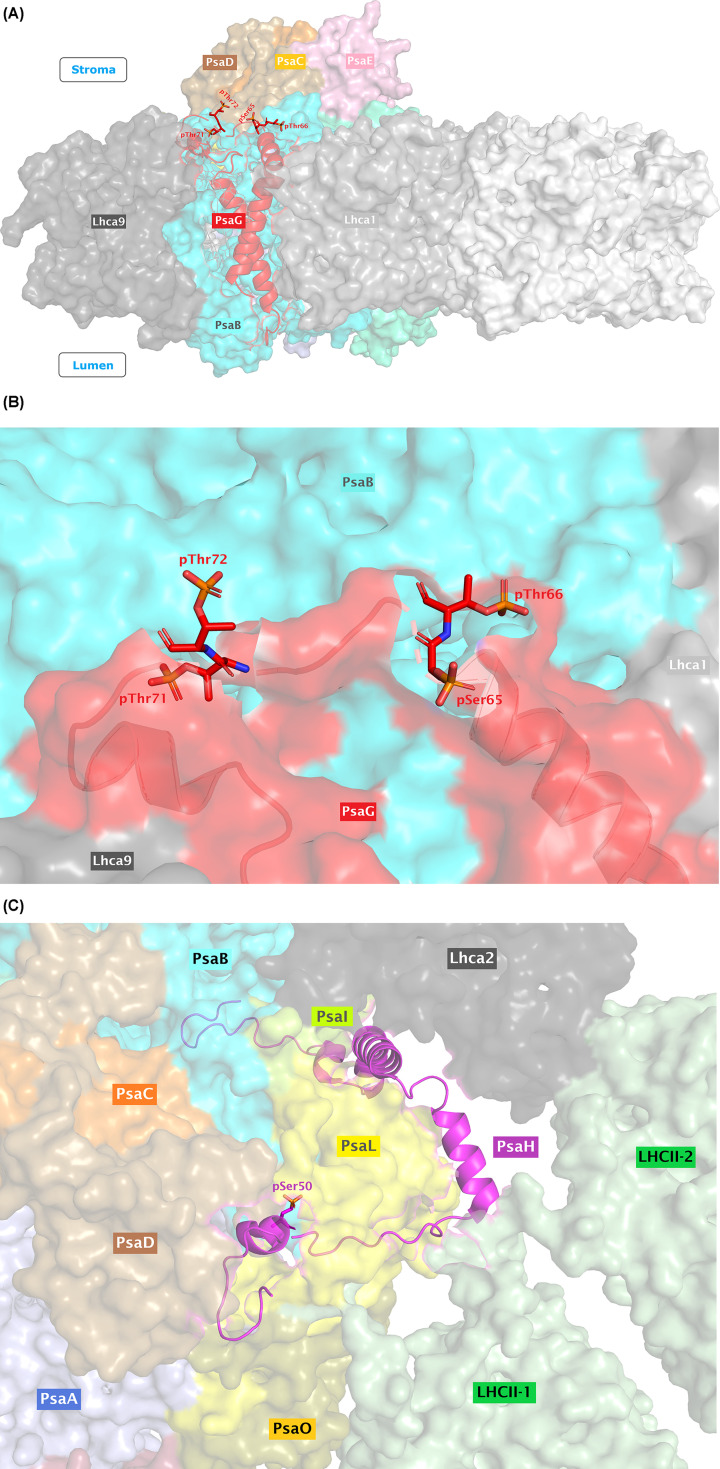
Phosphorylated sites in PsaG and PsaH subunits (**A**) An overview of PsaG phosphorylated sites using PSI dimer as a reference structure. For convenience, only one side of the PSI dimer is shown. The phosphorylated residues (pSer65, pThr66, pThr71, and pThr72) are represented in sticks. (**B**) A zoomed-in view of the phosphorylated residues (pSer65, pThr66, pThr71, and pThr72) in PsaG, as shown in sticks representation. (**C**) An overview of the phosphoresidue pSer50 in PsaH, as shown in stick representation, using the reference structure PSI–LHCI–LHCII (PDB ID: 7DZ7).

The potential impact of increased phosphorylation of PsaG (S65, T66, T71, and T72) and PsaH (S50) was further analyzed on a structural level. Figure 1A–C shows the structures of *C. reinhardtii* PSI-10Lhca (Monomer; PDB ID: 6JO5), PSI–LHCI–LHCII (state transition complex; PDB ID: 7DZ7), and PSI dimer (PDB ID: 7ZQD), respectively. The PsaG and PsaH subunits are highlighted in different representations. It is of note that the PsaH subunit is present in the state transition complex, while the PsaG is present in all of these structures. Contrastingly, the PsaH has also been observed in PSI–LHCI supercomplex (PDB ID: 6IJO) [14]; however, it is only partially resolved (residues 101–151) and therefore lack the phosphorylated residue pS50 as well as the structural residues interacting with LHCII trimers and the stabilized loop in PsaL. Similarly, the phosphorylated serine/threonine residues in PsaG and PsaH subunits are displayed in Figure 3A–C. For convenience, the phosphate groups have also been added to the residues of interest using PyTMs plugin for PyMOL.

### Phosphorylation of PsaH and stabilization of nearby loop in PsaL

**Figure 4:**
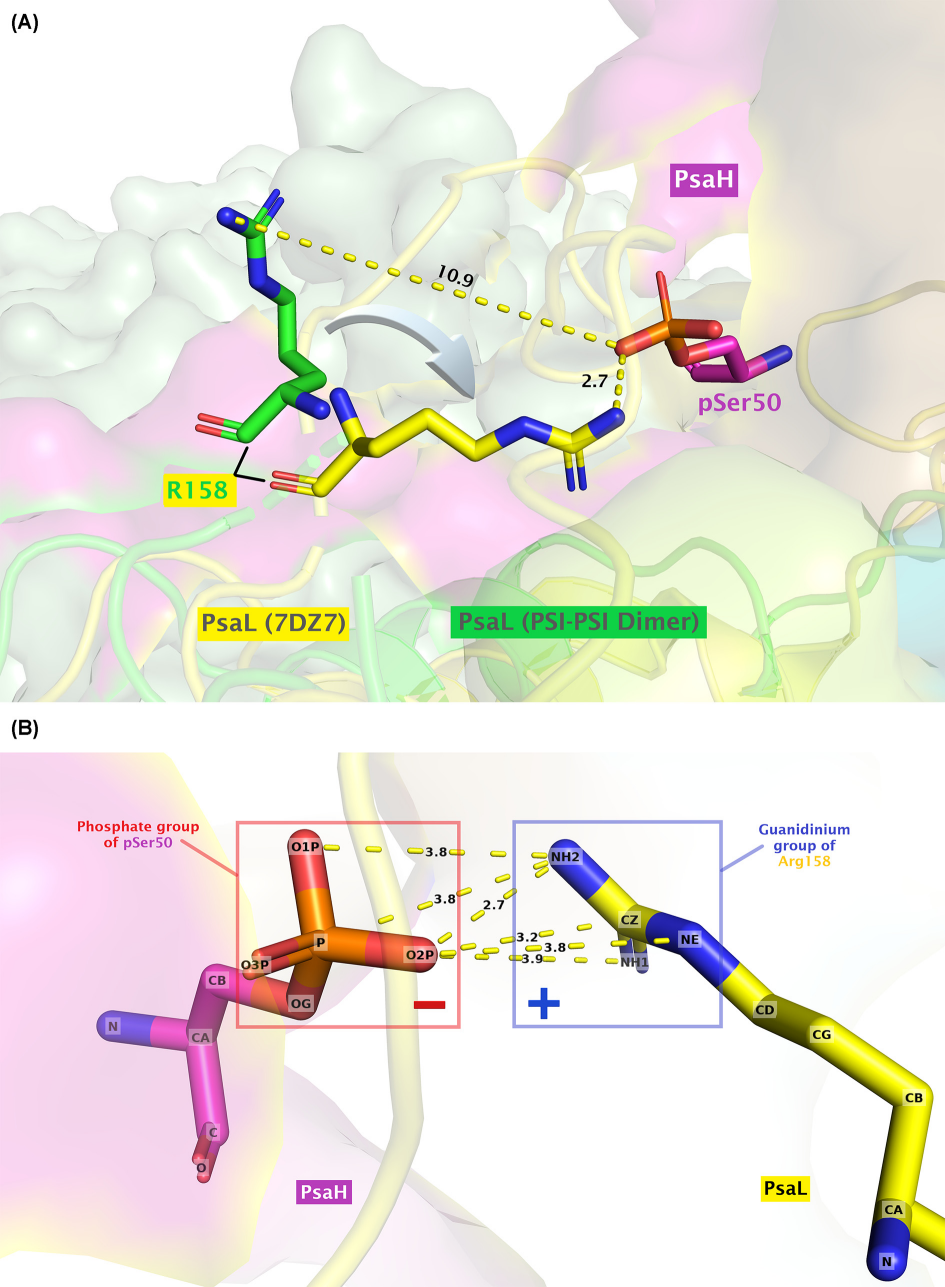
Potential functional role of PsaH phosphorylation at position 50 (pSer50) (**A**) Superposition of the dimeric PSI structure (PDB ID: 7ZQD) with PSI–LHCI–LHCII (PDB ID: 7DZ7) shows a tilting of R158 in PsaL of PSI–LHCI–LHCII toward the phosphorylated serine residue (pSer50) in PsaH. The PsaL subunits are shown in cartoon while the residues are displayed as sticks. (**B**) Distance between the atoms in the phosphate group of pSer50 and the guanidinium group of R158 within 4.0 Å limit.

Superposition of PsaL subunits from PSI dimer and PSI–LHCI–LHCII shows a clear tilting of R158 in PsaL subunit of PSI–LHCI–LHCII toward the pS50 in PsaH, thereby implying that this phosphorylation probably aids in stabilizing PsaH subunit in PSI (Figure 4A). Compared with lysine (which carries only a single amino group), arginine can make stronger and additional interactions with the phosphate groups due to the presence of the guanidinium side chain in Arg [59,60]. Moreover, Woods and Ferré [61] also suggested that electrostatic interaction between arginine and phosphate imparts a ‘covalent-like’ stability. Figure 4B shows the distance between the atoms in the guanidinium group of R158 in PsaL and the phosphate group of pS50 in PsaH within 4.0 Å threshold, as determined with PyMOL built-in ‘find contact’ option. Considering the distance between the phosphate group of pS50 in PsaH and the guanidinium group of R158 (shortest 2.7 Å), it can lead to the formation of a salt bridge with one oxygen atom of the phosphate group and/or attractive charge with the other oxygen atom, as predicted with Discovery Studio Visualizer. However, the actual orientation of the phosphate group in experimental model could vary, but still they are close enough to each other to qualify for such an interaction. Regarding the possible PsaH phosphorylation interference with the binding of the LHCII trimers to the PSI (due to the fact that negative–negative charges repel each other), the phosphorylated pS50 of PsaH seems very far (14.5 Å) from the phosphorylated residue pT27 of LhcbM1 of LHCII-1 in the state transition complex [25] (Supplementary Figure S1). Similarly, the R154 of PsaL, which is supposed to be interacting with the pT27 of LhcbM1 (distance 2.4 Å from each other), also does not seem to be disturbed with the phosphorylation of S50 of PsaH, since the relative position between these residues is also far (13.5 Å) from each other in the state transition complex (Supplementary Figure S1). This would support the view that phosphorylation of PsaH stabilizes its binding to PsaL and thereby to the PSI core complex.

**Figure 5:**
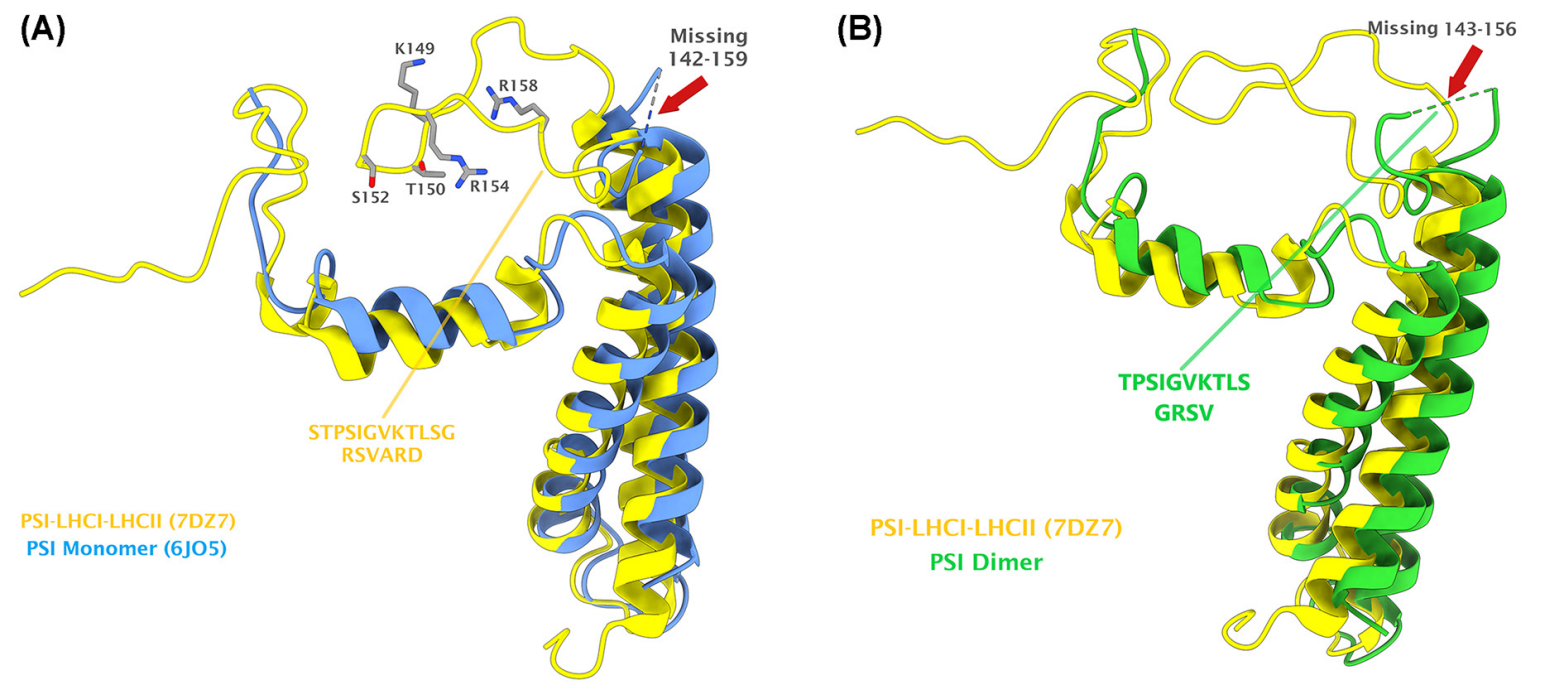
Stabilization of the loop in PsaL, which is involved in the interaction with pS50 in PsaH as well as pT27 in LhcbM1 (**A**) Superposition of the PsaL subunits from state transition complex (yellow) and the PSI monomer (blue). Red arrow indicates the missing residues (142–159) from monomeric PSI. The interacting partners that lie in this loop of PsaL in state transition complex are shown in gray sticks. (**B**) Superposition of the PsaL subunits from state transition complex (7DZ7) and PSI dimer (7ZQD). The missing residues (143–156) from PSI dimer are highlighted by the red arrow. Note that the R158 (unresolved in the monomer) is available in the dimeric PSI.

**Figure 6:**
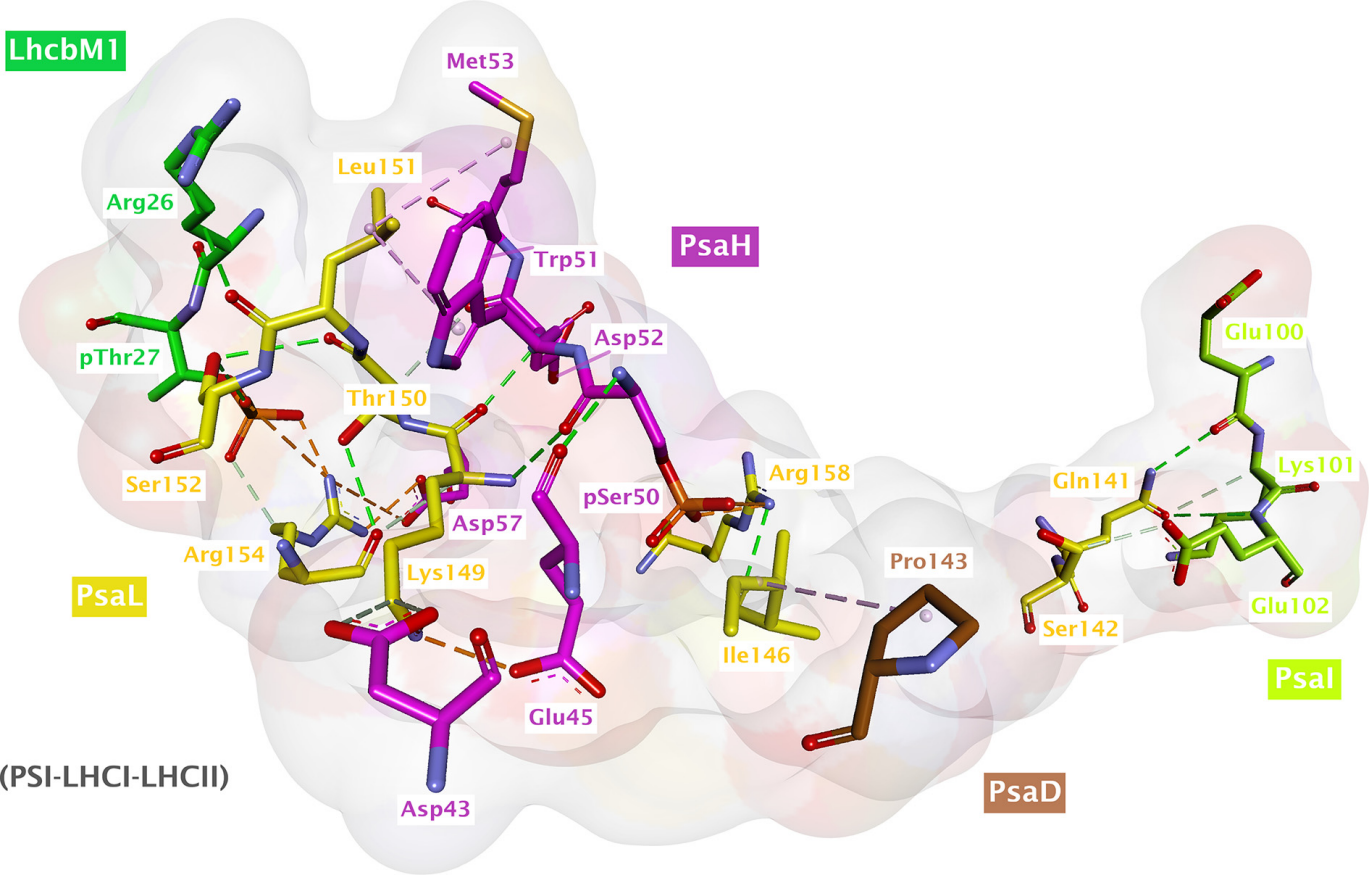
Interactions of residues from the stabilized loop in PsaL with the residues from neighboring subunits (PsaH, PsaI, PsaD, and LhcbM1) during state transitions (also summarized in Table 1) For convenience, only the residues involved in the interaction are shown.

Moreover, structural alignments indicate the stabilization of a loop (residues 142–159 in PSI monomer (PDB ID: 6JO5); 143–156 in PSI dimer) in PsaL near the pS50 of PsaH and pT27 of LhcbM1 during state transitions (Figure 5). Such a loop was found well-resolved in the state transition complexes (PDB IDs: 7DZ7 and 7D0J) but found distorted in the monomeric and dimeric PSI structures. Surprisingly, this loop of PsaL contains the key residues involved in stabilizing the PsaH as well as state transition complexes, such as residues K149 and R154. Though unresolved in the monomeric PSI structures, residue R158 has recently been resolved in the PSI dimer structure (PDB ID: 7ZQD), but it rather points away from pS50 of PsaH, as elaborated above. Figure 6 reveals the interaction of different residues from the newly stabilized loop of PsaL with the residues from nearby subunits (also summarized in Table 1). Residue R154 of PsaL has previously been found to interact with pT27 of LhcbM1 of LHCII-1 in the state transition complex as well as the nearby D57 of PsaH [25]. Detailed analysis with Discovery Studio Visualizer indicates that R154 can give rise to a salt bridge, an attractive charge and a carbon hydrogen bond, respectively, with O1P, O2P, and O3P atoms of the phosphate group of nearby pT27 of LhcbM1 (shortest distance: 2.42 Å). Similarly, D57 of PsaH, which lies at a very close distance of 3.04 Å from R154 of PsaL, also has the potential to make a salt bridge and an attractive charge with R154 of PsaL (Figure 6 and Table 1). Considering the possible interaction between K149 of PsaL and pS50 of PsaH, the side chain amino group of K149 is 6.8 Å apart from the phosphorylated residue (pS50) of PsaH and 10.9 Å away from the phosphorylated residue (pT27) of LhcbM1 in the state transition complex (Supplementary Figure S1). However, there are two closely associated negatively charged residues (D43 and E45) in PsaH that are, respectively, 4.3 and 3.3 Å apart from the amino group of K149 in PsaL. Therefore, this K149 seems to favor a salt bridge with E45 and also qualifies for attractive charge and carbon hydrogen bond formation with D43 of PsaH. Furthermore, K149 can also make conventional hydrogen bonds with D52 and pS50 in PsaH using its other chemical groups (Supplementary Figure S2). To summarize, these data suggest that phosphorylation of PsaH subunit at position S50 seems to enhance the binding of PsaH to PSI by mainly interacting with R158 of the stabilized loop of PsaL. However, the current available structures for the state transitions in *C. reinhardtii* indicate that a stable PSI–LHCI–LHCII complex can exist without phosphorylation of S50. Yet again, there is also a possibility that PsaH S50 could get phosphorylated later when a state II complex has already been formed, thereby further contributing toward the stability of PsaH under this transition. Another possible reason for the phosphorylation of PsaH S50 and stabilization of nearby loop in PsaL could be attributed to the prevention of PSI dimerization under HL, as the structural alignment between PSI–LHCI–LHCII and PSI dimer complexes reveal that they are mutually exclusive, as dimer formation would clash with the physical features of the state transition complex [27]. Nevertheless, further experimentation is demanded in this regard.

**Table 1:** Possible interactions of PsaL loop (residues 142–159) with the surrounding subunits, including the phosphorylated pSer50 of PsaH in state transitions (PDB file: 7DZ7)

Subunit 1	Contact residue 1	Subunit 2	Contact residue 2	Distance (Å)	Bond type	Remarks (if any)
PsaL	Ser142	PsaI	Glu102	3.46	Carbon Hydrogen bond	‒
PsaL	Ile146	PsaD	Pro143	5.40	Alkyl interaction	‒
PsaL	Lys149	PsaH	Glu45	3.34	Salt bridge	‒
PsaL	Lys149	PsaH	Asp43	4.17	Attractive charge	‒
PsaL	Lys149	PsaH	Asp43	3.27	Carbon–hydrogen bond	‒
PsaL	Lys149	PsaH	pSer50	3.10	Conventional hydrogen bond	‒
PsaL	Leu151	PsaH	Met53	4.87	Alkyl interaction	‒
PsaL	Leu151	PsaH	Trp51	5.13	Pi-Alkyl	‒
PsaL	Leu151	LhcbM1 (chain Z)	Arg26	3.20	Conventional hydrogen bond	‒
PsaL	Ser152	LhcbM1 (chain Z)	pThr27	2.77	Conventional hydrogen bond	‒
PsaL	Arg154	LhcbM1 (chain Z)	pThr27	2.42	Salt bridge with O1P	‒
PsaL	Arg154	LhcbM1 (chain Z)	pThr27	4.19	Attractive charge with O2P	‒
PsaL	Arg154	LhcbM1 (chain Z)	pThr27	3.15	Carbon–hydrogen bond with O3P	‒
PsaL	Arg154	PsaH	Asp57	3.04	Salt bridge	PsaL Arg154 could interact with both Asp57 and pThr27 simultaneously
PsaL	Arg154	PsaH	Asp57	4.68	Attractive charge	PsaL Arg154 could interact with both Asp57 and pThr27 simultaneously
PsaL	Arg158	PsaH	pSer50	2.7	Salt bridge and/or attractive charge	Depending on actual orientation of phosphate group in experimental model

### Phosphorylation and stabilization of loop in PsaG

**Figure 7:**
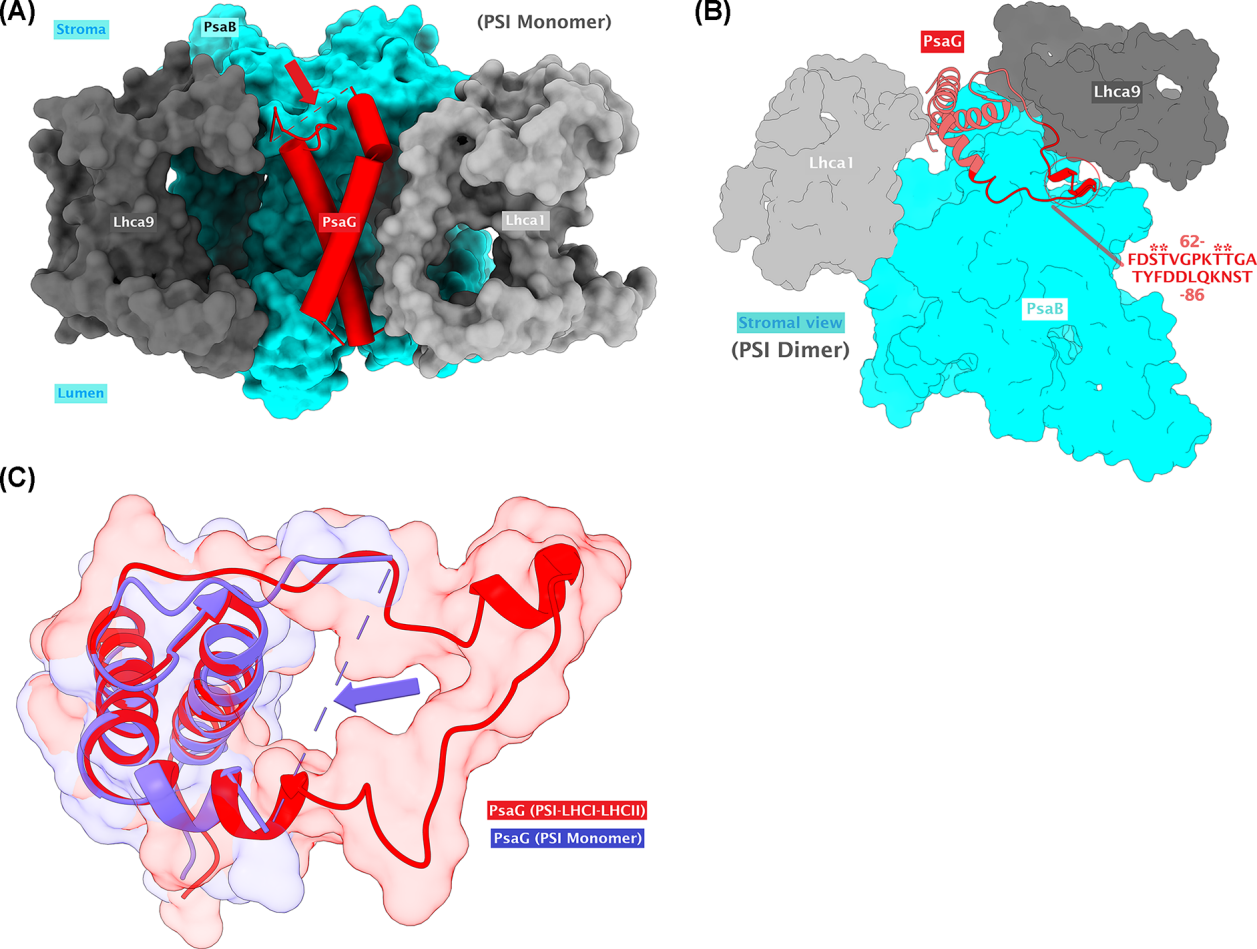
An overview of the distorted loop in PsaG that also contains the phosphorylated residues (**A**) A look into the missing loop region (indicated with the red arrow) in PSI monomer (PDB ID: 6JO5). (**B**) The loop (including encircled small helix; residues 63–85) is stabilized in the PSI dimer (PDB ID: 7ZQD). The phosphorylated residues are marked with asterisks. (**C**) Superposition of PsaG from PSI monomer (PDB ID: 6IJO) and state transition complex (PDB ID: 7DZ7) reveals the stabilization of the loop in the state transition complex as well.

**Figure 8:**
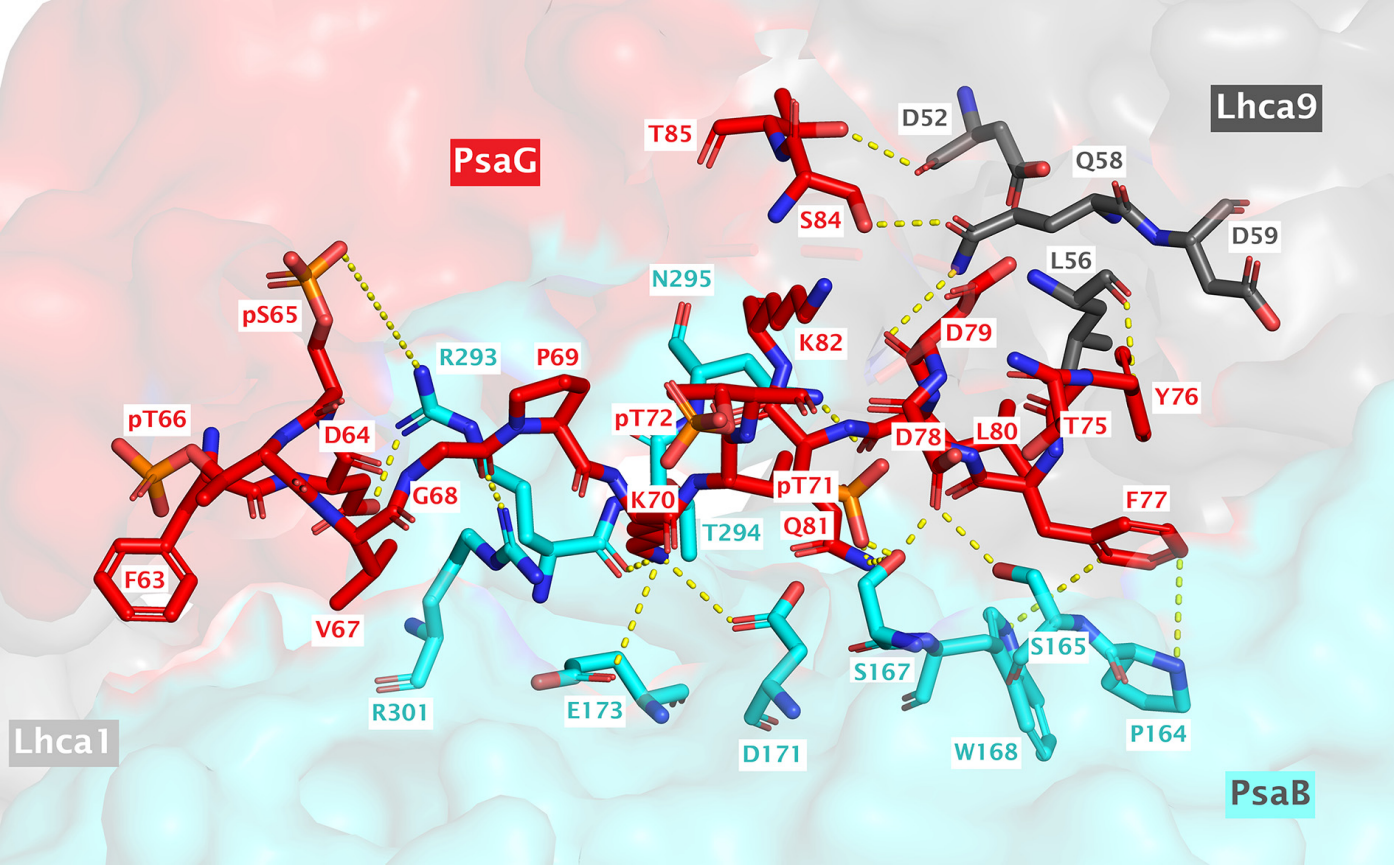
Interactions of PsaG residues (63–85) with the nearby residues from PsaB and Lhca9, using the PSI dimer as a reference structure (also summarized in Table 2)

Phosphoproteomic analysis identified the phosphorylation of four residues, namely pS65, pT66, pT71, and pT72, in the loop of PsaG located on the stromal side of PSI. Interestingly, this loop (residues 63–85), together with a short helix (encircled in Figure 7B), is also distorted in every monomeric structure of PSI available for *C. reinhardtii* but present only in the dimeric PSI and state transition complex (Figure 7A–C). Analysis of these residues (63–85) in the PSI dimer and state transition complex indicates that they further strengthen and stabilize the binding of PsaG to the PSI core as well as interacting with the nearby Lhca9 subunit, thereby improving its stability as well. Residues D52 and L56 of Lhca9 form conventional hydrogen bonds with T85 and Y76 of PsaG, respectively. Similarly, Q58 of Lhca9 also makes conventional hydrogen bonds with D79 and S84 of PsaG (Figure 8 and Table 2). On the other hand, majority of interactions could be identified between the residues of PsaB subunit with those from the stabilized loop region of PsaG (Figure 8 and Table 2). Of these, the key interactions were observed for F77 and K70 of PsaG. F77 of PsaG is a highly conserved residue (Supplementary Figure S3A) that forms Pi–Sigma (π–σ) bond as well as Pi–Pi (π–π) bond with P164 and W168 of PsaB, respectively (Supplementary Figure S4A,B). Additionally, F77 also makes π–π bond with the nearby Y76 of PsaG. Multiple sequence alignment indicates that P164 and W168 are also conserved across a wide range of species, while the Y76 is predominantly replaced with histidine (H) (Supplementary Figure S3B). K70, on the other side, interacts with D171, E173, and R301 of PsaB, leading to the formation of a salt bridge, an attractive charge and a conventional hydrogen bond, respectively (Supplementary Figure S4C,D). Similarly, among the phosphorylated residues of PsaG, pT71 seems to interact with S167 of PsaB. As far as the phosphorylation of S65 is concerned, two nearby potential interacting partners (R293 and R301) arise from the PsaB subunit (Figure 8 and Table 2). However, the distances between these residues vary when the state transition complex (7DZ7) is compared with the PSI dimer (7ZQD) (see Table 2 for distances). Nevertheless, since the addition of phosphate group in these structures is *in silico*, flexibility is expected to bring them even more closely together. Similarly, R293 of PsaB is also a highly conserved residue among various species whereas, on the other side, PsaB R301 is not that much conserved (Supplementary Figure S3C).

**Table 2:** Possible interactions of PsaG residues (63–85), including the phosphorylated amino acids, with PsaB and Lhca9 subunits using the state transition complex (7DZ7) and PSI dimer (7ZQD) as reference structures

Subunit 1	Contact residue 1	Subunit 2	Contact residue 2	Distance (Å)	Bond type	Remarks (if any)
PsaG	Phe63	PsaB	Ala304	4.5	Pi-Alkyl	Observed in 7DZ7 only
PsaG	Phe63	PsaB	Arg301	4.2	Pi-Alkyl	Observed in 7DZ7 only
PsaG	Asp64	PsaB	Arg293	3.0	Salt bridge; Attractive charge	Attractive charge in 7DZ7 (Distance 4.9 Å)
PsaG	Asp64	PsaB	Arg301	4.6	Attractive charge	Observed in 7DZ7 only
PsaG	pSer65	PsaB	Arg293	7.7	Electrostatic	Distance is 8.3 Å in 7DZ7
PsaG	pSer65	PsaB	Arg301	7.5	Electrostatic	Distance is 9.4 Å in dimer
PsaG	pThr66	PsaB	Lys321	6.4	Electrostatic	Observed in 7DZ7 only
PsaG	Val67	PsaB	Arg301	5.1	Alkyl	Observed in 7DZ7 only
PsaG	Gly68	PsaB	Arg301	2.1	Conventional hydrogen bond	‒
PsaG	Lys70	PsaB	Asp171	3.4	Salt bridge; Attractive charge	‒
PsaG	Lys70	PsaB	Glu173	4.3	Attractive charge	‒
PsaG	Lys70	PsaB	Arg293	2.9	Conventional hydrogen bond	‒
PsaG	pThr71	PsaB	Ser167	1.4	Polar contact	‒
PsaG	Tyr76	Lhca9	Leu56	3.4	Conventional hydrogen bond	‒
PsaG	Phe77	PsaB	Pro164	2.6	Pi–Sigma	Distance counted from aromatic ring centroid
PsaG	Phe77	PsaB	Trp168	5.7	Pi–Pi	Distance counted from aromatic ring centroid
PsaG	Asp78	PsaB	Ser165	2.8	Conventional hydrogen bond	‒
PsaG	Asp78	PsaB	Ser167	3.4	Carbon–hydrogen bond	Observed in 7DZ7 only
PsaG	Asp79	Lhca9	Gln58	3.7	Conventional hydrogen bond	‒
PsaG	Leu80	PsaB	Asn295	3.2	Conventional hydrogen bond	‒
PsaG	Gln81	PsaB	Ser167	3.5	Conventional hydrogen bond	‒
PsaG	Gln81	PsaB	Asn295	3.1	Carbon–hydrogen bond	Observed in 7DZ7 only
PsaG	Asn83	PsaB	Asn295	2.8	Carbon–hydrogen bond	Observed in 7DZ7 only
PsaG	Ser84	Lhca9	Gln58	3.0	Conventional hydrogen bond	‒
PsaG	Thr85	Lhca9	Asp52	3.1	Conventional hydrogen bond	‒

Another important aspect that has been observed is a possible electrostatic interaction between K321 of PsaB and the phosphorylated (pT66) residue of PsaG in the state transition complex, which are 6.4 Å apart from each other. However, the shortest distance between these two residues is 10.9 Å in the PSI dimer. Furthermore, superposition of PsaG from PSI dimer and the state transition complex reveals a change in conformation in some residues near the Lhca1 pole. Such a change is dramatic for the PsaG F63 and D64 (Supplementary Figure S5), subsequently giving rise to additional interactions with the PsaB subunit in state transition complex. Pi–Alkyl interactions, for instance, could be observed for F63 of PsaG with R301 and A304 of PsaB (Supplementary Figure S6) which, on the other side, are not evident in the PSI dimer. Interestingly, this is the region where the phosphorylated residues (pS65 and pT66) also occur. It is well-known that phosphorylation has the ability to modify the structural conformation of a protein [62,63]. Therefore, in this case, the changes induced in the structure of PsaG near the Lhca1 pole could be due to the phosphorylation of S65 and/or T66. However, as the loop is found distorted in every monomeric PSI, it is hard to ascertain the precise conformational changes associated with PsaG phosphorylation during these transitions. Notably, there is also evidence for association of one or two LHCII trimer to Lhca1 [64], which could be modulated via flexible phosphorylation of PsaG. As PsaG pT72 does not seem to be involved in interactions with Lhca9 or PsaB and points toward the stroma, this phosphorylation could also be involved in LHCII trimer binding toward the Lhca1 side. However, it is unclear whether the binding of LHCII trimer(s) to Lhca1 is independent from state transition complex formation or not. Moreover, interactions were also observed for F77 and L80 of PsaG with the nearby Chl (CLA) molecules (CLA811 and CLA812, respectively) in both PSI dimer and state transition complex (Supplementary Figure S7A). Interestingly, two new interactions with additional CLA molecules could be seen in the PSI dimer. F63 and D64 in the PsaG of PSI dimer can interact with CLA822 and CLA821, respectively (Supplementary Figure S7B), which seem to be disturbed in the state transition complex, probably due to the conformational change in PsaG loop, as elaborated previously. The fact that whether phosphorylation of these residues itself could result in stabilization of PsaG loop is difficult to judge for the moment because currently available state transition complex as well as the dimeric PSI structures reveal that such a configuration is possible and even stable without phosphorylation but there is also a probability that phosphorylation of these residues can take place once a complex is already been there and therefore the stability is enhanced further upon subsequent phosphorylation.

Previously, it has been shown that state transition in *C. reinhardtii* is induced by HL [65]. PsaG phosphorylation was found to be absent in a STT7 mutant of *C. reinhardtii* [26], thus indicating a link between PsaG phosphorylation and state transitions. On the other hand, in *C. reinhardtii*, phosphorylation of PsaH is STT7 independent [64] but also favors formation of the state transition complex according to the above structural considerations. In HL, phosphorylation of PsaH (S50) is about two times more up-regulated compared with HL-induced PsaG phosphorylation (Figure 2). It is of note that, at the same time, the abundance of PSI and PSII core proteins significantly diminish, including LhcbM proteins and their phosphorylation(s). The pronounced decrease of LhcbM phosphorylation, such as S242 of LhcbM3, could indicate that under HL less ‘classical’ state transition complexes are formed. At the same time, as mentioned above, phosphorylation of PsaH and PsaG significantly increases. This increase is embedded in the overall increase of N-terminal LHCSR3 phosphorylation. Particularly, phosphorylation of LHCSR3 S26 with concomitant phosphorylation of either S28, T32, or T33 was more than 26 times more abundant in HL than in NL (Figure 2). This is accompanied by a strong decrease of Lhcb4 T7 and T11 phosphorylation and a decrease in overall PSII abundance in accordance with [58]. Due to the strong increase in LHCSR3 abundance, HL cells are likely in a qE-promoting state as revealed previously [58]. In this regard, it is tempting to speculate that strong phosphorylation of PsaG and PsaH might tune PSI–LHCI for effective photoprotection, which apparently involves the stabilization of PSI–LHCI–LHCII complex, where LhcbM proteins bind to form the classical state transition complex [25] and/or associate with Lhca1 [64]. Likewise, association of LHCSR3 and LHCSR1 to these complexes will facilitate light to heat dissipation [66] under HL conditions. Absence of PsaH phosphorylation, on the other hand, might destabilize its binding to PSI and change conformation of PsaL. This might indicate that PSI dimer formation is regulated independently from state transitions and is present rather in low light, where its phosphorylation is significantly less abundant, in accordance to *Arabidopsis*, where closely packed PSI dimers were more abundant in the dark state [28]. In this scenario, nonphosphorylated PsaH would be more loosely attached to PSI, promoting unbinding of Lhca2 and PSI dimer formation. Although we based our argumentation on the grounds of available structural information, we cannot exclude that phosphorylation of PsaH and PsaG does also occur in the unassembled state. In regard of PsaG, there are no reports of unassembled PsaG in the thylakoid membrane. On the other hand, PsaH, in contrast with other PSI subunits, was found to be specifically enriched within the pyrenoid tubules in *C. reinhardtii* [34]. Thus, differential phosphorylation of PsaH could contribute to distinct localizations of the subunit, yet, functional data are missing.

## Conclusion

Conclusively, the PsaG and PsaH subunits of PSI in *C. reinhardtii* reveal dynamic phosphorylation in response to different light conditions. Structural analysis indicates that the phosphorylation of PsaH subunit at position S50 seems to be important for its stabilization to the PSI, ultimately facilitating the state transition complexes. On the other hand, phosphorylation of PsaG subunit appears to be important for the formation of both PSI dimer and state transition complexes due to stabilization of PsaG and Lhca9 to the PSI. Moreover, besides phosphorylation, stabilization of loops in PsaG and PsaL subunits appear to further aid these processes as they harbor several key residues engaged in either direct interaction with the phosphorylated residues or providing additional interacting partners for the stabilization of subunits necessary for the dimerization of PSI as well as formation of the state transition complexes in green algae.

## Supplementary Material

Supplementary Figures S1-S7

Supplementary Table S1

## Data Availability

Project Name: Phosphoproteomic analysis of the high light response of *Chlamydomonas reinhardtii* Project accession: PXD036166 Project DOI: 10.6019/PXD036166 Reviewer account details: Username: reviewer_pxd036166@ebi.ac.uk Password: zOZfkaXe

## Abbreviations

AGC: automatic gain control
BCA: bicinchoninic acid
Chl: chlorophyll
Cryo-EM: cryogenic electron microscopy
FASP: filter-aided sample preparation
FDR: false discovery rate
FDX: ferredoxin
FNR: ferredoxin-NADP-reductase
HCD: higher-energy C-trap dissociation
HL: high light
LB: loading buffer
LC-MS/MS: liquid chromatography–tandem mass spectrometry
LFQ: label-free quantification
LHCI: light-harvesting proteins of PSI
LHCII: light-harvesting proteins of PSII
MOAC: metal oxide affinity chromatography
MS: mass spectrometry
NADPH: reduced nicotinamide adenine dinucleotide phosphate
NL: normal light
PCY: plastocyanin
PDB: protein data bank
PMSF: phenylmethylsulfonyl fluoride
PSI: photosystem I
PSII: photosystem II
SDS: sodium dodecyl sulfate
TCEP: tris (2-carboxyethyl) phosphine
TFA: trifluoroacetic acid
TP: tris-phosphate
WT: wild-type
YFP: yellow fluorescent protein
