# Supplementary material for: Remodeling of algal photosystem I through phosphorylation
Source: Biosci Rep. 2023 Jan 24;43(1):BSR20220369. doi: 10.1042/BSR20220369 (PMC9874419; doi:10.1042/BSR20220369)
Supplement: Supplementary Figures S1-S7 [file BSR-2022-0369C_supp.pdf]

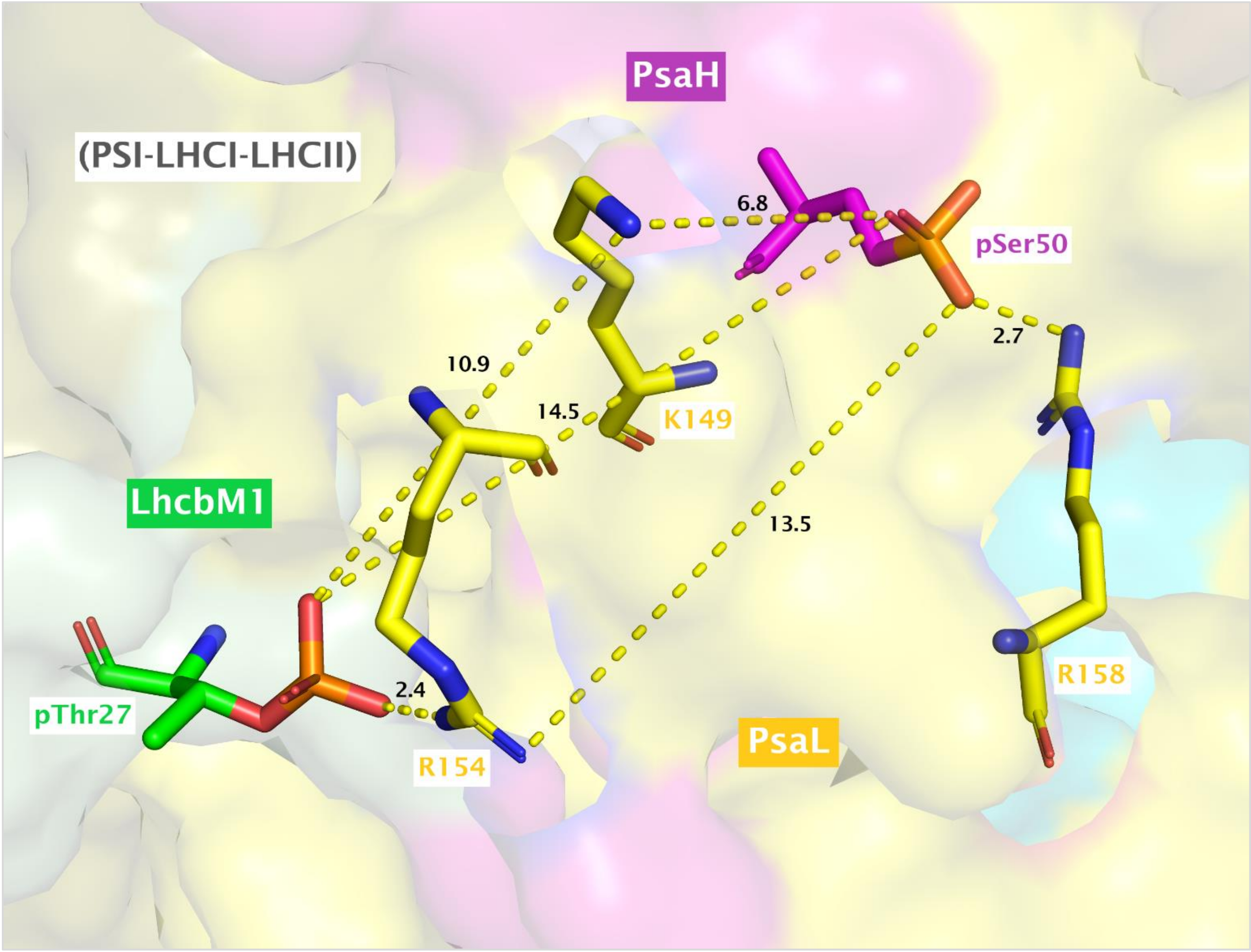

Supplementary Figure S1. pSer50 of PsaH and K149 of PsaL are far from pThr27 of LhcbM1. Also, pSer50 doesn't seem to interfere with R154 of PsaL, which is close to pThr27 of LhcbM1.

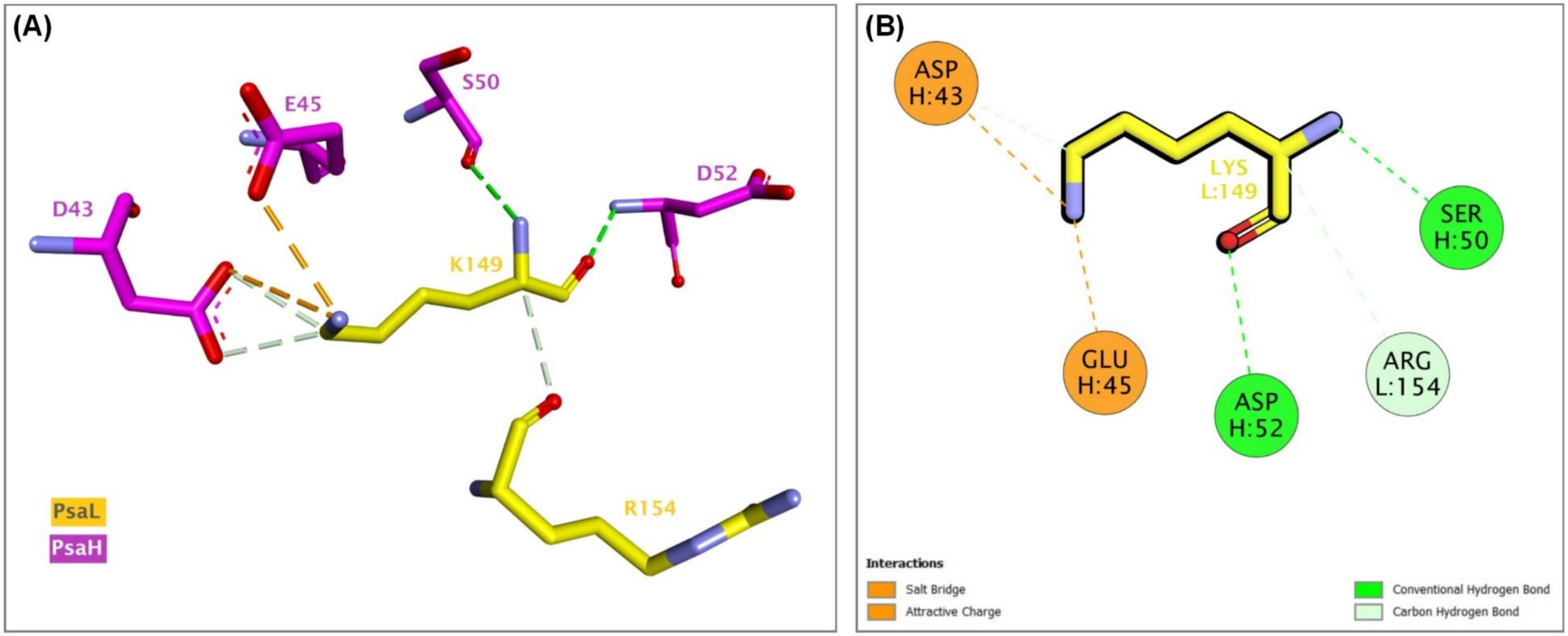

Supplementary Figure S2. Interactions of K149 from PsaL with the nearby residues from PsaH in the state transition complex. (**A**) 3D representation of K149 interactions. (**B**) 2D representation of K149 interactions.

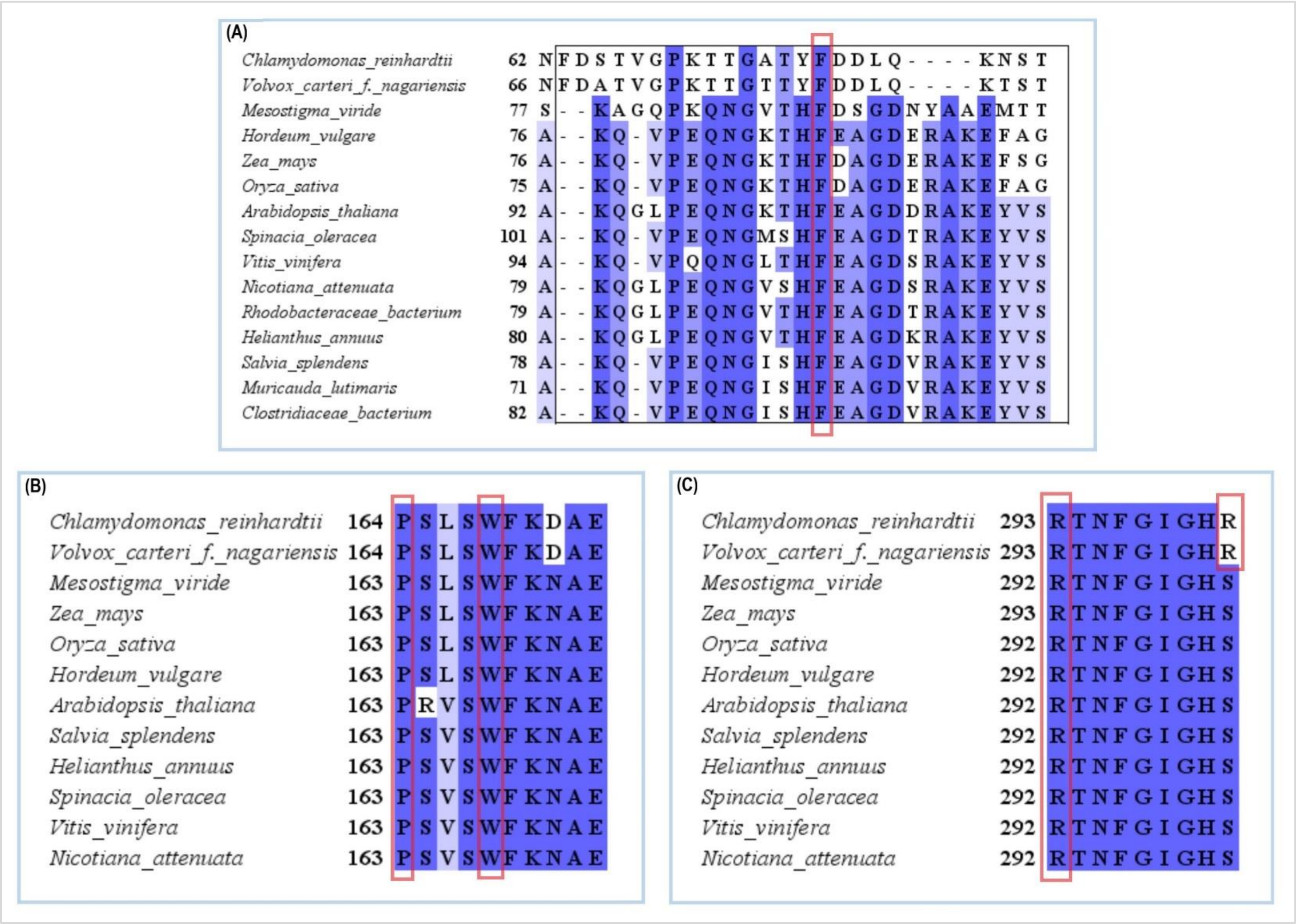

Supplementary Figure S3. Multiple sequence alignment of PsaG (residues 62-85) and PsaB (residues 164-173; 293-301) from various species. (**A**) F77 is highly conserved in PsaG. (**B**) P164 and W168 of PsaB, which interact with F77 in *Chlamydomonas*, are also conserved. (**C**) R293 of PsaB is also conserved among various species. Colored according to percentage identity. For convenience, only specific number of residues are shown in the alignment.

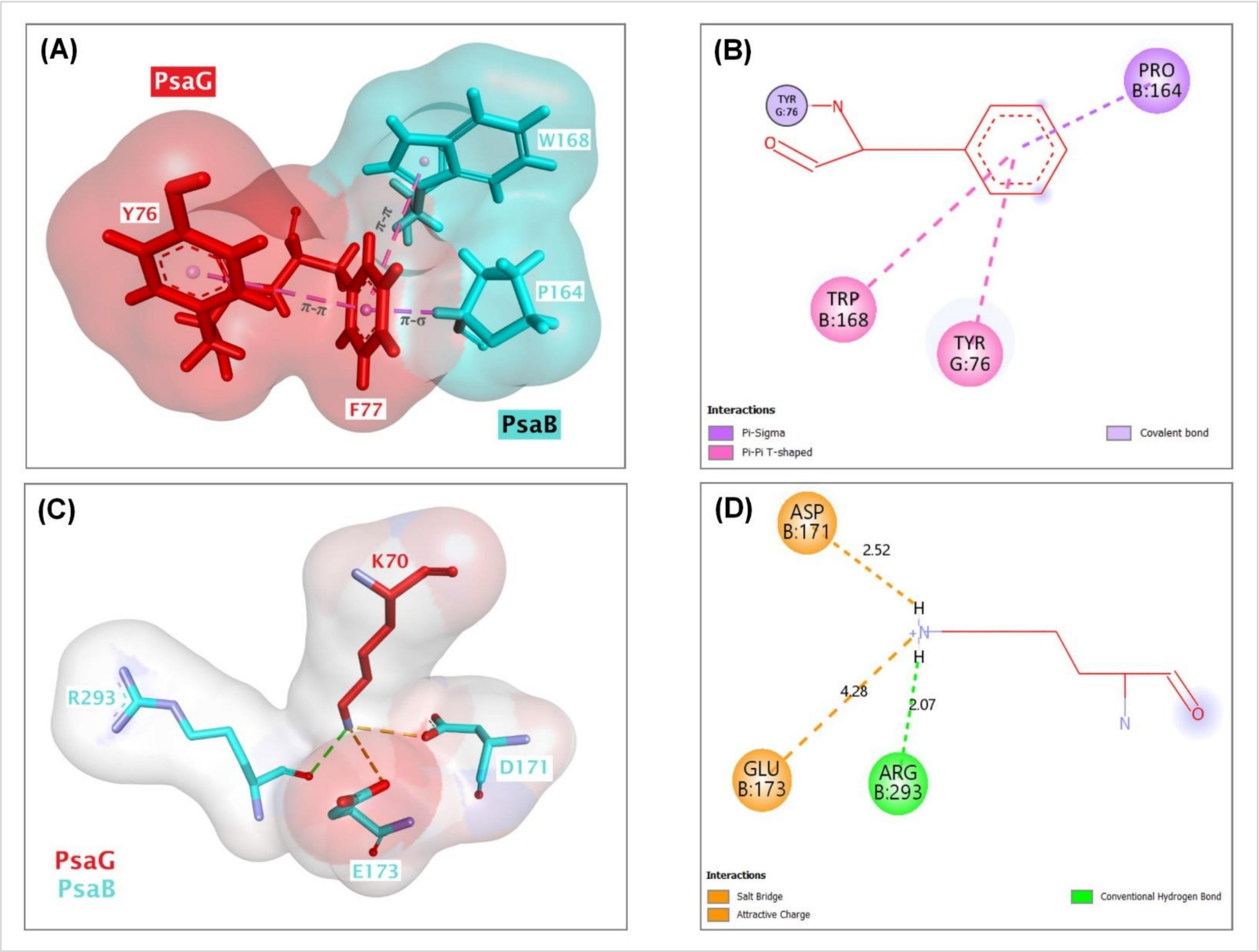

Supplementary Figure S4. (**A**) Interaction of F77 from PsaG with P164 and W168 of PsaB. (**B**) 2D representation of F77 interactions. (**C**) Interaction of K70 from PsaG with D171, E173 and R293 of PsaB. (**D**) 2D representation of K70 interactions.

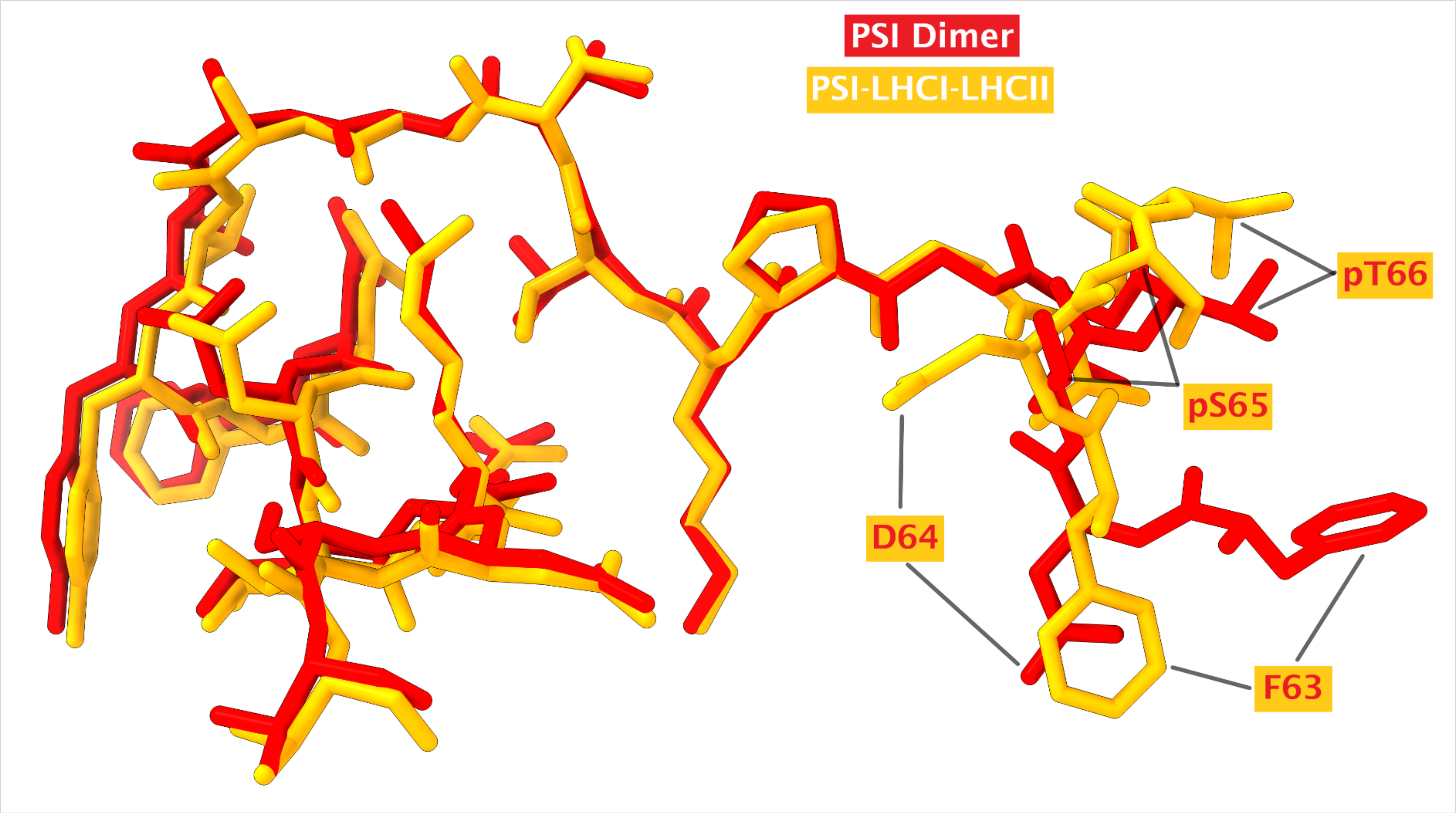

Supplementary Figure S5. Superposition of PsaG subunit residues (63-85) from PSI dimer (7ZQD) and state transition complex (7DZ7) show a change in conformation near the Lhca1 pole. Notice the change in positions of F63 and D64. The change in conformation of F63 leads to additional Pi-Alkyl interactions with R301 and A304 of PsaB subunit in 7DZ7 (Supplementary Figure S6). The nearby phosphorylated residues pS65 and pT66 are also highlighted.

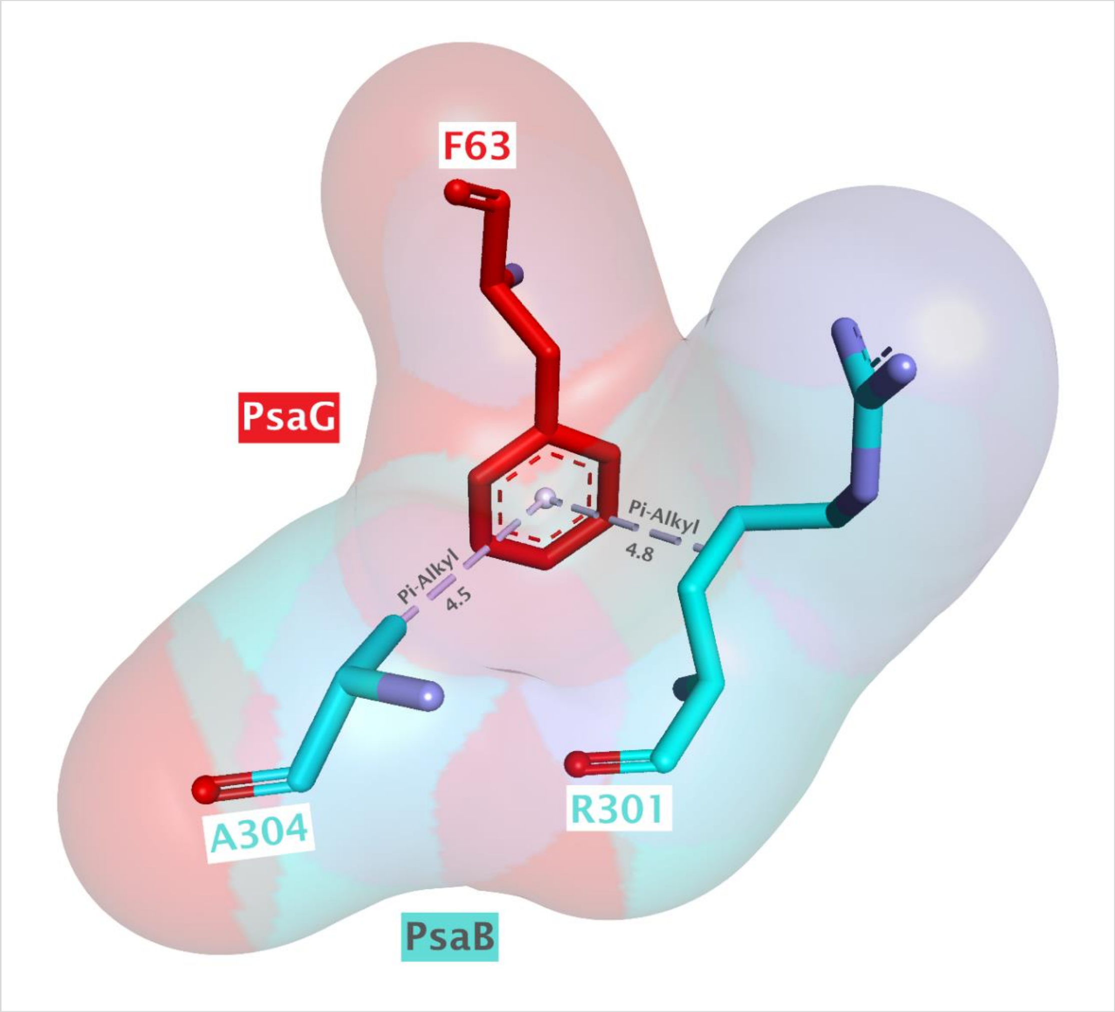

Supplementary Figure S6. Interaction of F63 from PsaG with R301 and A304 of PsaB in the state transition complex (7DZ7).

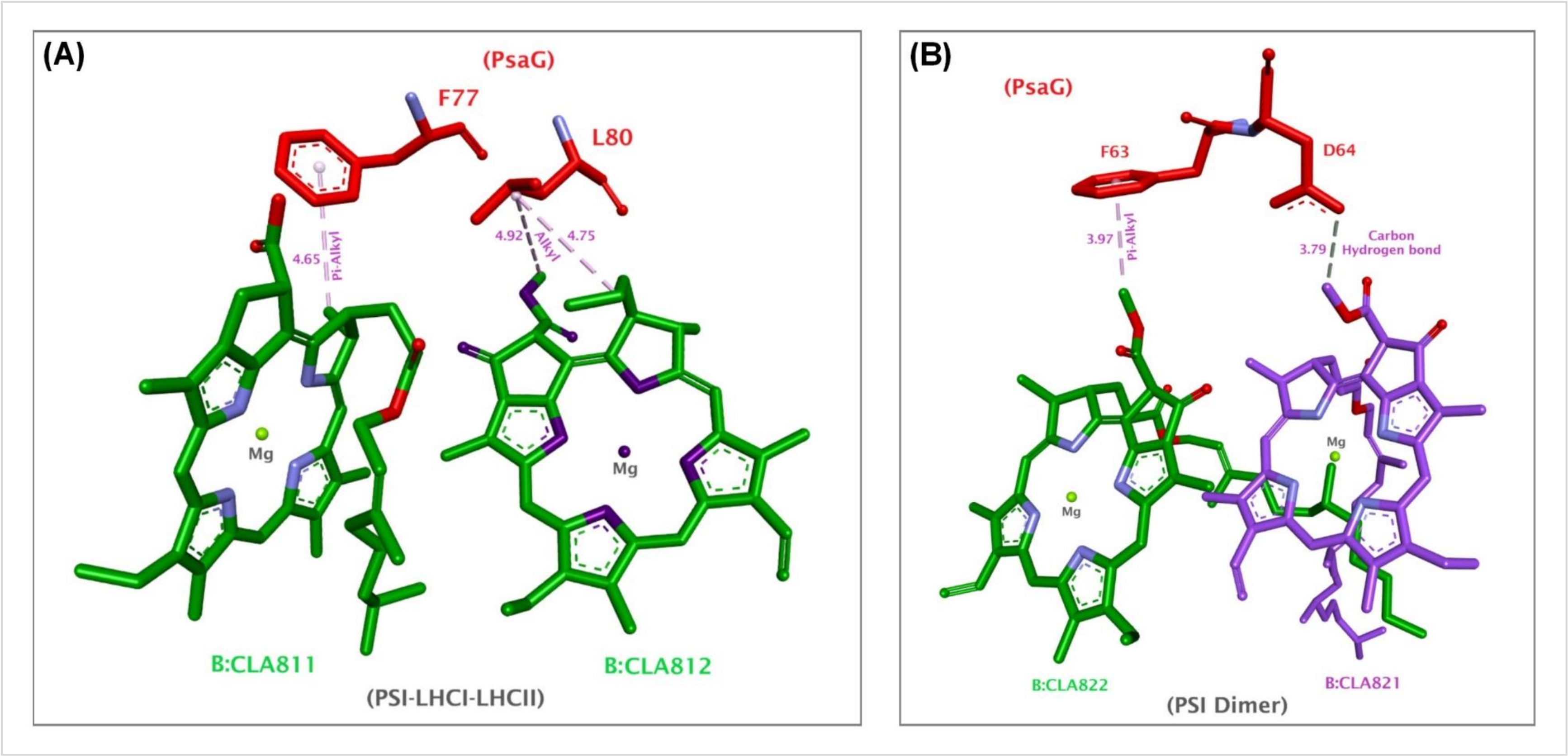

Supplementary Figure S7. Interactions of PsaG loop (residues 63-85) with chlorophyll molecules, as determined with Discovery Studio Visualizer. (**A**) Interaction of F77 and L80 with CLA811 and CLA812 in the state transition complex (7DZ7). Such interaction is found in PSI dimer as well. (**B**) Additional interaction of F63 and D64 with CLA822 and CLA821 in PSI dimer. These additional interactions are absent in the state transition complex due to conformational changes near the Lhca1 pole (Supplementary Figure S5).
